# A mixed-method of the happy-productive: wellbeing and performance patterns of remote workers in Brazil

**DOI:** 10.3389/fsoc.2025.1625831

**Published:** 2025-10-22

**Authors:** Amalia Raquel Pérez-Nebra, Esther Villajos, Jonathan Peñalver

**Affiliations:** ^1^University of Zaragoza, Zaragoza, Spain; ^2^IDOCAL, University of Valencia, Valencia, Spain; ^3^Valencian International University, Valencia, Spain

**Keywords:** happy-productive, mixed method, Brazil, remote work, emotions at work, job performance

## Abstract

The present study aims to examine the relationship between job wellbeing (emotions at work) and job performance (in-role, extra-role) and their connection in remote work using open questions. The sample consists of 297 Brazilian remote workers. Using a mixed-method approach, the findings reveal a 4-cluster pattern associated with the relationship between job wellbeing and job performance (i.e., 9-to-5, entrenched, engaged, and burned-out). Moreover, only high-performance patterns showed a relationship with the four categories of issues associated with remote work. Some issues are transversal to all groups, such as the Trade-off experience and the Adaptability process. However, Social exchange is only important for the entrenched pattern and the Lack of social resources is only linked to the engaged pattern.

## 1 Introduction

Although questions related to wellbeing at work have always been important for individuals and organizations (Schulte and Vainio, 2010), interest in this topic has increased since COVID-19 (Wong et al., 2020). Within organizations, interest in wellbeing at work is associated with a wide range of issues, such as intention to quit (Pelly, 2023), presenteeism (Jeong et al., 2020), absenteeism (Soriano et al., 2018), and job performance (Warr and Nielsen, 2018). In the literature, the relationship between wellbeing at work and job performance is commonly referred to as the happy-productive worker (Staw, 1986). In other words, there is a linear relationship: a happy worker is a productive worker, while an unhappy worker is an unproductive worker (Wright and Cropanzano, 2007). However, the historical review set out by Sender et al. (2020) indicates that research has been conducted since the 1920s to better understand the relationship between wellbeing at work and job performance, remains unclear.

Based on these results, Peiró et al. (2014) developed a proposal to address some of the questions surrounding this relationship: The Sustainable Productivity and Wellbeing Synergy (SPWS). The SPWS suggests a new approach to the happy-productive worker in two ways: (1) More comprehensive operationalization of the happiness and productive constructs; (2) Exploring the happy-productive relationship from a person-centered perspective. Using SPWS (Peiró et al., 2014) as a framework, some studies have expanded this relationship by proposing different variables (e.g., self-efficacy, Ayala et al., 2017; i-deal, Latorre et al., 2021; human resources practices, Tordera et al., 2020; work design, Pérez-Nebra et al., 2022). However, there is still much to be discovered. For example, new variables that focus on current organizational issues (i.e., experiences associated with remote work, Beckel and Fisher, 2022; Pérez-Nebra A. R. et al., 2021), integrating quantitative and qualitative data (i.e., mixed-method; Gibson, 2017), and extending the research to include non-WEIRD (Western, Educated, Industrialized, Rich, and Democratic) samples such as Brazil (Pitesa and Gelfand, 2023).

Therefore, the present work has a threefold aim: (1) to explore the relationship between workplace wellbeing (i.e., positive and negative emotions) and job performance (i.e., in-role, ex-role), through a person-centered approach. (2) To qualitatively explore how people cope with working remotely and what factors contribute to positive experiences; (3) To relate the job wellbeing/performance interaction to experiences associated with remote work.

### 1.1 Happy-productive worker

Happy workers perform better than unhappy workers. This is the idea behind the happy-productive worker thesis (Staw, 1986). However, as noted by Sender et al. (2020), the thesis is not precisely new, which also implies the existence of certain limitations (e.g., lack of consensus on the operationalization of the terms happiness and productivity, Sender et al., 2020). Moreover, despite the consensus on the positive (weak) relationship between happiness and productivity, some authors have revealed a non-linear relationship. In other words, not all happy workers are high performers (Baron et al., 2012; Grant and Schwartz, 2011), and sometimes even low levels of happiness can result in high performance (Silvestro, 2002). Recently, several proposals have emerged to address this issue. This is the case of the theory of Peiró et al. (2014), called the Sustainable Productivity and Wellbeing Synergy (SPWS). The SPWS is a heuristic approach to the happy-productive worker thesis, focused on the person, instead of on variables. SPWS is defined as the promotion and maintenance of the synergy of happy workers who show high levels of job performance and the search for a mutually reinforcing connection between wellbeing and productivity. Specifically, the SPWS (Peiró et al., 2014) could be described in three statements: (1) Happiness is understood as a fusion of hedonic and eudaimonic wellbeing; (2) Productivity considers variables such as in-role, ex-role, and creative performance; (3) four profiles result from the interaction between job wellbeing and job performance: happy-productive, unhappy-unproductive, happy-unproductive, and unhappy-productive. Several studies conducted following this approach have revealed promising results. Through the study of job satisfaction and innovation in a sample of young Spanish workers, Ayala et al. (2017) found that psychological contract, personal initiative, job self-efficacy, and over-qualification allowed to distinguish between the different happy-productive worker profiles. Furthermore, Tordera et al. (2020) studied the effects of human resource practices on the likelihood of belonging to each profile, considering factors such as employee age, the impact of i-deals (Latorre et al., 2021), and the influence of work design (Pérez-Nebra et al., 2022). Building on these results and the SPWS (Peiró et al., 2014), it has been found that different profiles can also be found at a group level (Peñalver et al., 2023), suggesting a possible homology process (i.e., equivalent structural relations across levels of analysis; Guenole, 2016).

Despite the progress made to understand the happy-productive worker thesis fully, there are still unanswered questions. First, our understanding of what defines workers within each profile is still in its early stages (i.e., Ayala et al., 2017; Abdi et al., 2019; Latorre et al., 2021; Peiró et al., 2019, 2021; Pérez-Nebra et al., 2022; Tordera et al., 2020). Although previous studies have proposed some personal and organizational characteristics, more job characteristics should be analyzed. In fact, recent events such as COVID-19 (Wong et al., 2020) may suggest variables about what employees consider relevant to explain the interaction between job wellbeing and job performance. Second, Pitesa and Gelfand (2023) noted that most organizational research has been conducted with Western, Educated, Industrialized, Rich, and Democratic individuals, also called WEIRD samples. This implies a significant knowledge gap as it neglects emerging countries like those in the BRICS group. This fact also refers to the results based on SPWS (e.g., Tordera et al., 2020) mentioned before, given that the data are from a Spanish sample.

About Brazil, findings on the happy-productive worker are still ongoing. On the one hand, a systematic review made with Brazilian studies revealed that in-role and ex-role performance shows an unclear relationship with job wellbeing, particularly when wellbeing is operationalized as hedonic (e.g., financial job satisfaction; Pérez-Nebra A. et al., 2021). On the other hand, a previous study conducted with a sample of Brazilian educational workers revealed a 4-cluster/profile solution: happy-productive, happy-unproductive, unhappy-productive and unhappy-unproductive (Latorre et al., 2021; Pérez-Nebra et al., 2022). The authors pointed out that the specific combination of task, social, and contextual characteristics in the workplace was related to the likelihood of belonging to each of the different profiles. Considering these arguments, we propose:

*Hypothesis 1 (H1):* A 4-cluster solution will emerge in a sample of workers in Brazil.

### 1.2 Remote working

As discussed above, many contexts and job characteristics may still affect workers’ wellbeing and performance. A recent development that has emerged is working remotely (Ng et al., 2022). Remote work or telework is defined as working outside of a conventional office setting, such as at home or in a remote office, using information communication technology for communication and work tasks (Beckel and Fisher, 2022). Given its direct impact on working conditions, teleworking has specific particularities that can change the relationship with work and make us (un)happier and more (or less) productive (Eurofound, 2022). In other words, although the relationships between remote work, job wellbeing, and job performance have been studied, contradictory results have been found.

In terms of job wellbeing, numerous articles suggest a positive relationship between telework and job wellbeing, in particular regarding health (Beckel and Fisher, 2022), affective wellbeing (Anderson et al., 2015), lower stress levels (Delanoeije and Verbruggen, 2020), or job satisfaction (Erro-Garcés et al., 2022). However, a positive relationship has also been found with professional isolation (Golden et al., 2008) and poorer mental health and quality of life (Mendonça et al., 2022). Regarding job performance, previous research has found that telework has an important and positive effect on performance (Vega et al., 2014). However, a meta-analysis by Martin and MacDonnell (2012) suggests that remote working has a positive (although small) relationship with different indicators of performance (like productivity or retention). For instance, telework positively affects employees’ turnover intentions (Nemțeanu and Dabija, 2023). In sum, these positive or negative relationships may depend on work-life balance, support from the organization/supervisor, or the perception of work control, home office constraints, work uncertainties, and inadequate tools (Ipsen et al., 2021). Also, full-time telework showed the lowest levels of employee wellbeing compared to partial telework, occasional telework and having some degree of telework ability, which reported the highest level of wellbeing (Eurofound, 2022).

Aligned with the results found, the advantages of remote work, such as work-life balance, work efficiency, and work control, as well as the disadvantages, such as home-office constraints, uncertainties and tools, are likely to emerge as variables that influence the job wellbeing and job performance relationship.

To further explore how such factors emerge in employees’ own words and to capture unanticipated dimensions, we adopted an innovative and inductive mixed-method approach, leading to the following exploratory hypothesis:

*Hypothesis 2 (H2):* In a remote work context, keywords reflecting advantages and disadvantages of remote work will emerge from participants’ narratives.

Although remote workers report advantages and disadvantages of working from home, they are different from each other. They have different perceptions and different needs. Thus, we focused on employee voice to compare the keywords that emerged within each profile. Existing literature indicates that clusters tend to be coherent with their discourse; in other words, qualitative and quantitative analyses tend to converge (e.g., Mishima-Santos et al., 2021). Therefore, the four clusters are expected to report different perceptions of advantages and disadvantages. We propose:

*Hypothesis 3 (H3):* The positive profile (happy-productive) would report the advantages of remote work, and the most negative (unhappy-unproductive) would report the disadvantages of remote work.

## 2 Method

### 2.1 Sample and procedure

Using a snowball sampling technique, an online questionnaire was shared through organizations’ intranet and social media. A total of 566 valid questionnaires were collected from Brazilian workers. However, considering the remote working framework, only participants with any experience working from home were considered and answered the qualitative questionnaire. This means they work from home for at least 10% of the week. Thus, the final sample comprised 297 workers (59.2% females; mean age 42.5 years, SD = 9.6). The average tenure in the company was 13.6 years (SD = 8.35), and 66.0% had completed a master’s degree. Workers are white-collar, in the public sector, and mainly public servants (64.66%).

### 2.2 Variables and instruments

#### 2.2.1 Wellbeing at work

We applied an emotion at work scale (original, Segura and González-Romá (2003); adaptation into Brazilian-Portuguese, Paschoal and Tamayo, 2008). The scale is composed of positive (e.g., “At my job I feel… optimistic”) and negative (e.g., “At my job I feel… nervous”) emotions at work and has good reliability (available in Table 1) (χ^2^/*df* = 0.83; CFI = 1, TLI = 1; RMSEA = 0.00 CI90% = [0.00–0.06]). Participants were asked to rate each item on a 5-point scale, ranging from 1 (totally disagree) to 5 (totally agree), considering the agreement with each statement based on their current work.

**Table 1:** Means, standard deviations, reliability, and correlations for the study variables.

Variables	M	SD	K	S	α	ω	1	2	3
1. Positive emotion	2.80	1.14	−0.51	−0.04	0.95	0.95	–	–	–
2. Negative emotion	2.94	1.10	−0.50	−0.13	0.90	0.90	−0.29**	–	–
3. Extra role performance	4.58	1.55	−0.62	−0.52	0.82	0.85	0.15**	0.11*	–
4. Intra role performance	5.33	1.17	1.59	−1.12	0.88	0.89	0.34**	0.01	0.33**

M, Mean; SD, Standard Deviation; K, Kurtosis; A, Skewness; α, Cronbach alpha; ω, Omega index.

#### 2.2.2 Job performance

We used the scale of Goodman and Svyantek (1999) with two dimensions of performance: Extra-role performance (3 items, item example: “Helps other employees with their work when they have been absent”) and Intra-role performance (3 items, item example: “Fulfils all the requirements of the job”). We adapted the scale to the Brazilian population, translating each item 4 times. We used a group of 12 experts (2 Ph.D., 3 Ph.D. students, 5 master’s degree students, and 2 undergraduate students) to judge which item “sounds” better for Brazilian workers following the decentering procedure (Smith et al., 2013) proposed by Pérez-Nebra et al. (2023). Therefore, the response options consisted of a five-point scale from 1 (totally disagree) to 7 (totally agree). Job performance showed good reliability (available in Table 1) (χ^2^/*df* = 4.05; CFI = 0.98, TLI = 0.96; RMSEA = 0.10 CI90% = [0.06–0.14]).

### 2.3 Open questions

Semi-structured questions were added at the end of the questionnaire. The questions aimed to describe facilitators and barriers of wellbeing and performance in remote work. We asked two different questions to push both contents, one positive (“Describe the positive aspects of working from home”) and the other negative aspect (“Describe the negative aspects of working from home”). Answering the questions was optional. Most workers answered the open question (*N* = 271; 91.24%).

### 2.4 Control variables

We controlled sex (0 = male, 1 = female, 2 = non-binary), age in years, the state of Brazil, educational level, telework time (the percentage of time working from home), and tenure in the organization.

### 2.5 Data analysis

To test the hypotheses, a two-step multi-method procedure was used.

#### 2.5.1 Step 1: preliminary analysis, cluster analysis and control variable

We conducted assumptions, reliability and descriptive and correlation analyses before performing the cluster analysis with performance and wellbeing. There were assumption violations; Intra-role Performance with left kurtosis (5.63). However, the visual inspection showed a mostly normal curve (Field et al., 2012). Next, we conducted the subsequent step of the analysis.

The 297 workers were clustered based on the four variables (Kent et al., 2014). Even though k-means distance is the most common, this study used Clustering Large Application (CLARA), which is used to deal with larger data (Pandya, 2017). For the person-centred approach, we tested the best number of clusters. According to the different approaches on the optimal number of clusters, different indicators (WSS, Silhouette, and GAP) suggest 3–6. For parsimonious and theoretical reasons, we decided to use 4 clusters. Each cluster achieved the minimum sample size required for cluster analysis and its comparison (Dalmaijer et al., 2022). Descriptive statistics were conducted to get an accurate picture of the clusters.

Finally, a one-way ANOVA was conducted with age and telework time to examine the difference in the ratio of belonging to any cluster. No differences were found.

#### 2.5.2 Step 2: lexical analysis

We added all the narratives and organized the corpus. We did it by standardizing the Portuguese language and connecting keywords. For example, telework, work from home, and work remotely had to be rewritten as telework and the name of the company or similar (e.g., in Bank Y, in the bank) was replaced with “organisation”; SarsCovid-equivalent was replaced with Covid. Finally, we also corrected some spelling mistakes. The lexical analysis used the Iramuteq (R interface) software and the Camargo and Justo (2018) Iramuteq protocol. We analysed 934 text segments, 5.07% occurrences and 47.4% forms of hapax. We also conducted Reinert Classification with Descendent Hierarchical Classification (DHC), which emerged 4 classes of words. Also, to compare the 4 lexical analysis classes between the 4 clusters (i.e., Lexical Analysis comparison), we conducted a chi-square analysis. Differences are considered significant when the test is greater than 3.84, based on 1 degree of freedom and *p* < 0.05. In this case, none of the 4 classes of words revealed significant differences.

## 3 Results

Table 1 presents the means, standard deviations, internal consistencies (Cronbach’s alpha and omega reliability coefficients), and bivariate correlations for all the study variables. The scales present acceptable reliability, and correlations were below 0.34.

**Figure 1:**
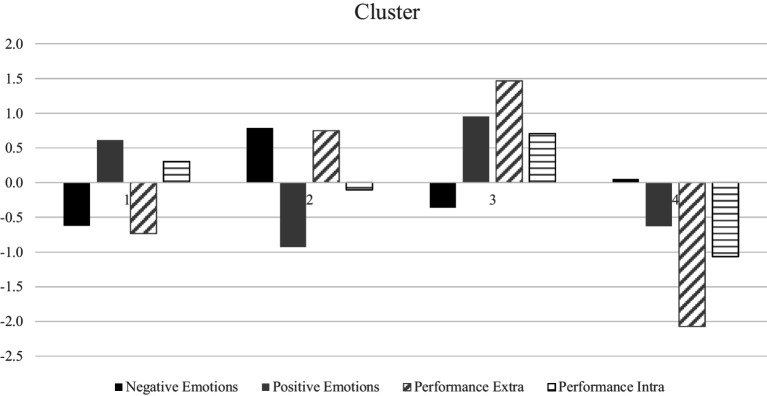
Plot of means across clusters.

A four-cluster solution was identified in the analysis. Specifically, Cluster 1 includes 34.34% (*N* = 102) of the sample, Cluster 2 includes 25.92% (*N* = 77), Cluster 3 includes 20.88% (*N* = 62) and Cluster 4 includes 18.86% (*N* = 56). The result pointed to a 4-cluster solution. Figure 1 shows the standardized mean of the cluster predictor.

The cluster analysis recommended the following interpretation of each cluster profile: Cluster 1: Just 9-to-5 (happy-just-productive), Cluster 2: Entrenched-workaholic (unhappy-productive), Cluster 3: Engaged (happy-productive), and Cluster 4: Burned-out (unhappy-unproductive). It is important to note that the personal and labour variables do not increase the likelihood of belonging to any cluster.

**Figure 2:**
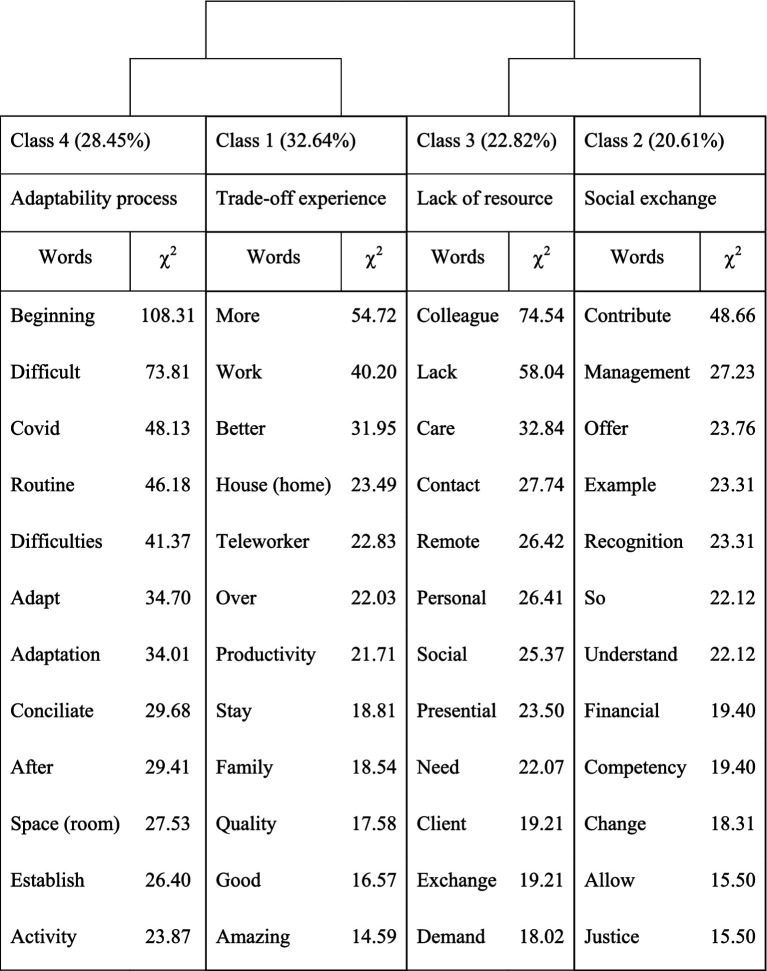
Dendrogram of the Descendent Hierarchical Classification (DHC).

Concerning the lexical analysis, the DHC grouped words into four classes based on the narratives, with some connections between classes 1 and 4, and classes 2 and 3 (Figure 2). The four identified classes were: Class 1: Trade-off experience (example of a typical segment of text: “… despite the workload, it was very comfortable to work from home, it was good to be able to be close to the family more often when everyone in the family was together at the same time.”); Class 2: Social exchange (e.g., “Especially when there is no financial recognition or support for it.”); Class 3: Lack of -social- resources (e.g., “I missed the face-to-face contact with my colleagues.”); Class 4: Adaptability process (e.g., “at first it was a bit difficult but then I adapted very well and today I prefer teleworking”). In Supplementary material, some examples of representative discourses for each category are provided.

**Figure 3:**
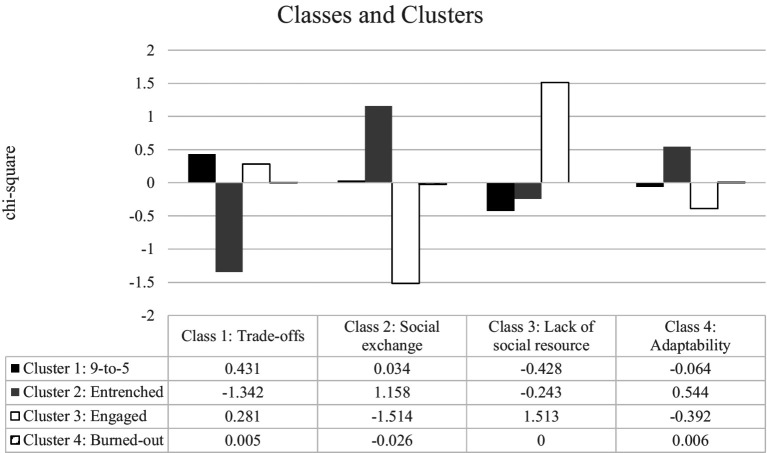
Chi-square between clusters and classes of lexicons.

Figure 3 combines qualitative and quantitative analyses. It is possible to notice that having no significant differences between groups only happened when considering *χ*^2^ less than 3.84 for a 0.05 tail; however, for a 0.25 tail, the cut-off is 1.32 and classes 1 to 3 were significantly different. Class 1, trade-off experiences, is less frequent for the entrenched cluster (Cluster 2). Class 2, the social exchange experiences, is more frequent for the entrenched cluster (Cluster 2) and less frequent for the engaged cluster (Cluster 3). Class 3, the perception of a lack of social resources, is more frequent in the engaged cluster (Cluster 3).

## 4 Discussion

The present work aims to investigate Sustainable Productivity and Wellbeing Synergy (SPWS) patterns in a sample of Brazilian teleworkers and describe the relationship between those patterns and remote work issues. We tested the relationship between job wellbeing and job performance by developing four profiles supporting H1. In addition, we examined the lexicon to determine if the keywords and variables identified and suggested in WEIRD samples are consistent in Brazil. Our findings revealed that different types of lexicons emerge, contradicting H2. Finally, we compared the clusters and lexicon, which presented a mixed result (H3). The entrenched and the engaged groups, who share positive performance, showed different lexicons; the entrenched pattern showed more social-exchange words, and the engaged pattern showed fewer social-resource words. Burned-out and 9-to-5 clusters presented no difference between each other and the other profiles.

The results of these profiles support the existence of four distinct profiles (H1), in line with previous studies conducted in Brazil (Latorre et al., 2021; Pérez-Nebra A. et al., 2021; Pérez-Nebra A. R. et al., 2021; Pérez-Nebra et al., 2022). However, some questions arise. The results from all these studies use a high-educated sample. Therefore, it is not clear if the result is sample-dependent or if it is a transversal finding. It is beyond the aim of this work, but it remains an open question.

The lexicon analysis presented four-word classes, which differ from what was expected (H2). The advantages and disadvantages suggested by the international literature seem to be only partially applicable to Brazil (Pérez-Nebra A. et al., 2021; Pérez-Nebra A. R. et al., 2021). Advantages such as work-life balance are interpreted more as a challenge in combination with home-office constraints. Work efficiency and work control do not emerge in this sample. This could be because those questions are not considered important or part of the employee agenda, and could be more related to managers. Disadvantages such as uncertainties and tool issues were also not the case, one explanation is the high-educated sample. However, other issues emerge, such as how to adapt and the lack of resources, particularly social resources, and social exchange, such as social recognition. Those questions are somewhat new and uncommon in the remote work literature, underscoring the contribution of the mixed-methods research approach.

Finally, the comparison of profiles with the lexicon analysis (H3) showed that the clusters with less productive profiles, namely the 9-to-5 and burned-out clusters, were less distinguishable compared to the other two. This could have different explanations. One explanation could be that they feel they are less allowed to express themselves. If they think they contribute less, they might believe that they cannot complain. Another explanation is that they do not want to express themselves because it may have negative consequences or, ultimately, because they think it is useless. On the other hand, entrenched workers do not refer to trade-offs, just social exchange (such as the need for recognition and financial compensation), and engaged workers do not express the need for social exchange but only the lack of social resources. The fact that the sample was in Brazil could explain the need for social resources and (social) recognition. As a collectivist country, social networks, relationships, and social and group issues are important. In more individualistic countries and samples, these issues may be less important and, therefore, not emerge as a theme. Interestingly, adaptability as a process emerges as an issue in remote work, but it is transversal across groups.

### 4.1 Theoretical and practical implications

The article presents some theoretical and practical implications worth mentioning. First, it advances the happy-productive worker thesis in several ways: (1) Applying the Sustainable Productivity and Wellbeing Synergy (SPWS, Peiró et al., 2014) to the happy-productive thesis (Staw, 1986) has allowed us to unravel relationships that are much more complex than theory would suggest. It is, therefore, confirmed that there may be a relationship beyond happy-productive (Sender et al., 2020), specifically for Brazilian workers. In fact, using the Brazilian sample has served to respond to the demands of previous research to use samples beyond WEIRD countries (Pitesa and Gelfand, 2023); (2) As noted by other authors (e.g., Ayala et al., 2017), more variables could play a significant role in the happy-productive worker thesis. For example, by considering the positive and negative emotions as wellbeing variables beyond job satisfaction, which implies a broader picture of job wellbeing conceptualization. Also, by adding telework issues as a job characteristic. Whether employees can telework may directly affect how wellbeing and performance relationships appear (Wong et al., 2020).

Second, thanks to the qualitative study, we were able to analyse in detail the reasons why teleworking places employees in each of the profiles, thus confirming previous research that suggests teleworking can have both positive (Erro-Garcés et al., 2022; Martin and MacDonnell, 2012) and negative (Mendonça et al., 2022; Nemțeanu and Dabija, 2023) relationships with the different outcomes. According to the TOE model, organizations must be able to provide a solid technology infrastructure for employees (Ng et al., 2022), they should also support and assist teleworkers to ensure their wellbeing or performance is not affected (Lamprinou et al., 2021), and the required country-level characteristics must be in place for everything else to function. In fact, some of these things have been found in the qualitative analysis: being able to have control or autonomy over the timetable and tasks, the lack of social contact, and how difficult it can be to adapt at the beginning. For instance, social-exchange, particularly some sort of recognition, emerges as an important variable and is new in this literature.

Concerning practical implications, first, a continuous psychosocial evaluation is crucial in organizations, to make decisions on time and create healthy job environments (Salanova et al., 2012). Second, the results guide companies in developing good teleworking practices to enhance wellbeing and sustainable performance. For example, support from the organization (Lamprinou et al., 2021) or manager (such as remote leadership, Ng et al., 2022) could be a key variable for teleworkers to be part of the happy-productive profile. Work-life balance practices could also be promoted, given that not only can teleworking help wellbeing and performance, but it is also important that workers can have autonomy over schedules, processes or management. For example, if remote working is not voluntary, it can be problematic for the employee. In addition, it is important to recognize the importance of organizations investing in good technology to facilitate access and daily work for employees who work remotely. This is necessary so that employees can telecommute effectively without reducing performance levels.

### 4.2 Limitations and future studies

The present study has several limitations. A first limitation is that a non-probabilistic sample (i.e., snowball sampling) was used, which might restrict the generalizability of these findings. However, requirements were established to ensure the reliability of the responses through inclusion criteria (i.e., working remotely in Brazil). Also, the study used a heterogeneous sample because it includes different companies (i.e., public service institutions, bank trade unions, private companies), which allows us to obtain a comprehensive view of the work reality.

Second, data were obtained from self-report measures, which might have caused common method bias. However, different response scales were used (5-point, 7-point, open questions; Podsakoff et al., 2003) to solve this issue. On the other hand, given the nature of this study, which includes psychological experiences such as emotions and remote work, it is difficult to use objective data.

Finally, there is yet another methodological limitation to operationalising what teleworking means. The questions asked of participants in both, the description of time spent teleworking and in the qualitative question, generically said “teleworking.” It also has limitations in comparing working from home with a co-working space. However, in the Brazilian context, teleworking is fundamentally working from home. Few people have the option to telework from shared offices. While in Europe it is common to find co-working spaces, in Brazil this is not yet a reality.

## Data Availability

The data that support the findings of this study are available from the corresponding author, upon reasonable request.
