# Supplementary material for: A mixed-method of the happy-productive: wellbeing and performance patterns of remote workers in Brazil
Source: Front Sociol. 2025 Oct 22;10:1625831. doi: 10.3389/fsoc.2025.1625831 (PMC12586098; doi:10.3389/fsoc.2025.1625831)
Supplement: Supplementary file 1 [file Table_1.DOC]

Supplementary Material

# Supplementary Figures and Tables

Table 1 represents some examples of representative discourses for each category. Iramuteq provides these verbal accounts. They contain a weighting, in descending order, of the most representative utterances for each word class. The most typical reports are presented here for illustration. These reports, combined with the lexicons, helped us to name each lexical class.

Table 1. Typical text segment per class of words

| Classes |  | Typical segment of text |
| --- | --- | --- |
| 1 | Trade-off experience | despite the workload it was very comfortable to work from home it was good to be able to be close to the family more times when everyone in the family was together at the same time  I thought it was good because I could control my schedule and do other activities, but I felt I worked a lot more and got more tired |
| 2 | Social exchange | Especially when there is no financial recognition or support for it.  […] financial compensation for a management position like the others who hold a management position |
| 3 | Lack of -social- resource | I missed the face-to-face contact with my colleagues.  I missed the face-to-face interaction with colleagues, it's important for the development of the work. |
| 4 | Adaptability process | at first it was a bit difficult but then I adapted very well and today I prefer teleworking  in the beginning it was difficult, but today I'm already well adapted I have to organize the tasks organization and discipline accumulation of functions because I can reconcile with the care of the children |
